# 

**Published:** 1908-10-10

**Authors:** 


### BOOKS RECEIVED.

The Clarendon Press, Oxford.
"An Alabama Student and other Biographical Essays." By Wm. Osler, M.D.

J. Nisbett and Co.
"Common Affections of the Liver." By W. Hale White, M.D.

J. Thinn, Edinburgh.
"A Guide to the Feeding of the Infant during the First Year." By J. W. Simpson, M.D.

J. Wright and Co., Bristol.
"A Synopsis of Surgery." By E. W. Hey Groves, M.D.

John Bale, Sons and Danielsson, Ltd.
"On Means for the Prolongation of Life." Third edition. By Sir Hermann Weber, M.D.

The Health Resorts Bureau.
"The Health Resorts of Europe." By Thomas Linn, M.D.

Rebman, Ltd.
"The Sexual Life of our Time." By Ivan Bloch, M.D. Translated from the German by M. Eden Paul, M.D.

Society for Promoting Christian Knowledge.
"The Tithe in Scripture." By H. Lansdell, D.D.

### ANSWERS TO CORRESPONDENTS.

*E. M. B.*—(1) Warwick and Tunstall's "First Aid," 1s., should suit your purpose. Heath's "Minor Surgery" and Pye's "Surgical Handicraft" may be a little too advanced. (2) "Elementary Dispensing Practice," 3s. 6d., by J. Ince.

### The Graduates' Medical Schools.

DIARY FOR WEEK OCT. 12 to OCT. 17.

### THE POST-GRADUATE COLLEGE, West London Hospital, Hammersmith, W.

**At 4.30 p.m.**
**Oct. 13,** Sir R. Douglas Powell, **Opening Address.**
**At 12.15 p.m.**
**Oct. 14 and 16,** Dr. Pritchard, **Practical Medicine.**
**At 5 p.m.**
**Oct. 14,** Dr. Beddard, **Medicine—I.**
**Oct. 15,** Mr. Baldwin, **Surgery—I.**
**Oct. 16,** Dr. Seymour Taylor, **The Treatment of Typhoid Fever.**

### NORTH-EAST LONDON POST-GRADUATE COLLEGE, Prince of Wales's General Hospital, Tottenham, N.

**At 4.30 p.m.**
**Oct. 13,** Dr. G. P. Chappel, **Demonstration of Selected Medical Cases.**
**Oct. 15,** Mr. Walter Edmunds, **The Treatment of Tuberculous Glands.**

### ST. JOHN'S HOSPITAL FOR DISEASES OF THE SKIN, Leicester Square, W.C.

**At 6 p.m.**
**Oct. 15,** Dr. Dockrell, **Essentials in the Study of Dermatology.**

### THE WEST-END HOSPITAL FOR NERVOUS DISEASES, Welbeck Street, W.

**At 5 p.m.**
**Oct. 15,** Dr. Harry Campbell, **Selected Cases.**

### MEDICAL GRADUATES' COLLEGE AND POLYCLINIC, 22 Chenies Street, W.C.

**At 5.15 p.m.**
**Oct. 12,** Dr. F. J. McCann, **How to Diagnose Cancer of the Womb.**
**Oct. 13,** Dr. G. H. Savage, **Borderlands between Badness and Madness.**
**Oct. 14,** Mr. C. R. B. Keetley, **Affections of the Toes, Feet, and Ankles.**
**Oct. 15,** Dr. Dundas Grant, **Remarks on Purulent Nasal Discharges.**

### NATIONAL HOSPITAL FOR THE PARALYSED AND EPILEPTIC, Queen Sq., Bloomsbury, W.C

**At 3.30 p.m.**
**Oct. 13,** Dr. Batten, **Infantile Paralysis.**
**Oct. 16,** Dr. Batten, **Family Diseases.**

